# PRMT5 promotes cancer cell migration and invasion through the E2F pathway

**DOI:** 10.1038/s41419-020-02771-9

**Published:** 2020-07-24

**Authors:** Wojciech Barczak, Li Jin, Simon Mark Carr, Shonagh Munro, Samuel Ward, Alexander Kanapin, Anastasia Samsonova, Nicholas B. La Thangue

**Affiliations:** 1 Laboratory of Cancer Biology Department of Oncology, University of Oxford, Old Road Campus Research Building, Oxford, OX3 7DQ UK; 2 Argonaut Therapeutics Ltd Magdalen Centre, Oxford Science Park, Oxford, OX4 4GA UK; 3 Centre for Genome Bioinformatics, St. Petersburg State University, St. Petersburg, 199034 Russia

**Keywords:** Colon cancer, Focal adhesion

## Abstract

The pRb-E2F pathway is a critical point of regulation in the cell cycle and loss of control of the pathway is a hallmark of cancer. E2F1 is the major target through which pRb exerts its effects and arginine methylation by PRMT5 plays a key role in dictating E2F1 activity. Here we have explored the functional role of the PRMT5-E2F1 axis and highlight its influence on different aspects of cancer cell biology including viability, migration, invasion and adherence. Through a genome-wide expression analysis, we identified a distinct set of genes under the control of PRMT5 and E2F1, including some highly regulated genes, which influence cell migration, invasio and adherence through a PRMT5-dependent mechanism. Most significantly, a coincidence was apparent between the expression of PRMT5 and E2F1 in human tumours, and elevated levels of PRMT5 and E2F1 correlated with poor prognosis disease. Our results suggest a causal relationship between PRMT5 and E2F1 in driving the malignant phenotype and thereby highlight an important pathway for therapeutic intervention.

## Introduction

E2F is a family of master transcription regulators involved in mediating diverse cell fates^1^. The retinoblastoma protein (pRb)-E2F pathway is a central factor in the control of cell cycle progression and its deregulation of primary importance in cancer, where aberrant pRb activity occurs through a variety of oncogenic mechanisms^1^. In the classical view, cyclin-dependent kinases, which peak during the G1 phase, phosphorylate pRb, causing the release of E2F, which then transcriptionally activates target genes required for cell cycle progression^2–5^. E2F1 is one of the most important physiological targets for pRb and the physical interaction between pRb and E2F1 facilitates transcriptional repression and cell cycle arrest^1,2^. Although E2F1 is able to foster diverse biological outcomes^6–8^, we continue to have a poor mechanistic understanding of the key pathways through which its influence is mediated.

In previous studies, we identified arginine (R) residues in E2F1 as a target for methylation by protein arginine methyltransferase 5 (PRMT5)^9,10^, enabling PRMT5 to prompt cell growth by influencing the E2F1 pathway^10^. The meR E2F1 mark is read by the tudor domain protein, p100/TSN^10^, which exists as a chromatin-bound symR E2F1 complex on E2F target genes^10,11^. Furthermore, among the E2F family, PRMT5-dependent methylation is uniquely relevant to E2F1^9,10^, suggesting that PRMT5 is fundamental to the control of E2F1 activity.

Here we have explored the functional role of the PRMT5-E2F1 axis. We found, using tumour cells where the *E2F1* gene had been genetically inactivated combined with chemical ablation of PRMT5 enzyme activity, that E2F1 is important for PRMT5 to maintain cancer cell viability. At the genome-wide expression level, we identified distinct sets of genes regulated by PRMT5 and E2F1 activity, with a specific set co-regulated by both PRMT5 and E2F1. Enriched Gene Ontology (GO) terms connected with cell motility were evident in the expression data. Further, PRMT5 and E2F1 regulate invasion and migration, and in human tumours we identified a coincidental expression between PRMT5, E2F1 and motility-related genes. In general, high levels of PRMT5 and E2F1 occurred in a range of cancers. Our results show for the first time that PRMT5, in part through the PRMT5-E2F1 axis, influences tumour cell migration and invasion, and further suggest that this relationship is significant in human disease.

## Results

### Effect of PRMT5 influenced by E2F1

**Fig. 1:**
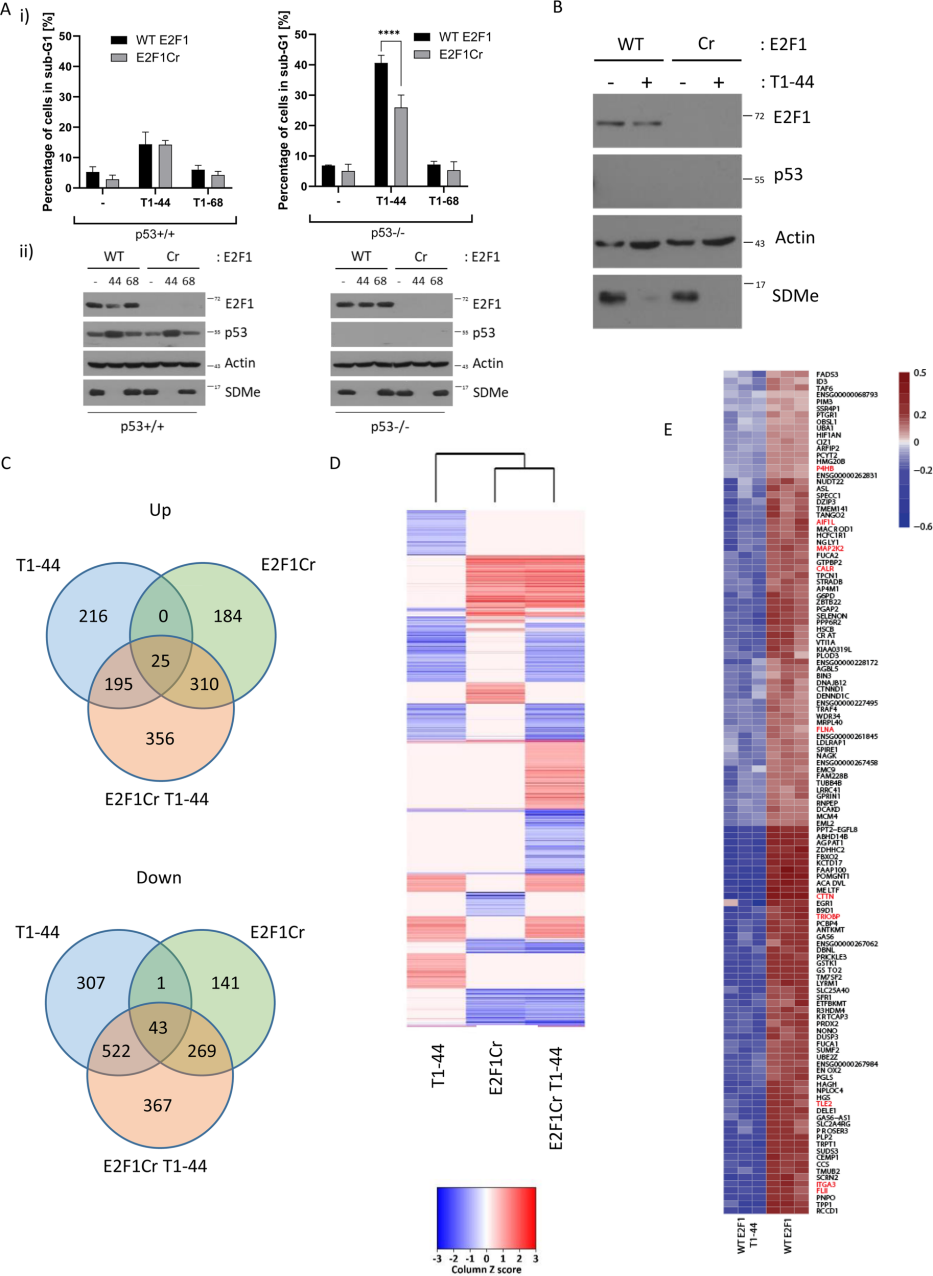
Genome-wide effects of PRMT5 inhibition dependent on E2F1. **a** Sub-G1 fraction analysis of WT E2F1 p53^+/+^ or p53^−/−^ HCT116 cells and E2F1 Cr p53^+/+^ or p53^−/−^ HCT116 cells, treated for 6 days with 1 μM PRMT5 inhibitor (T1–44) or less active compound (T1–68) (i). (ii) A representative example of an immunoblot is included to demonstrate the input protein levels of E2F1, p53 and symmetric arginine methylation (SDMe); actin included as a loading control. *n* = 3. **b** An immunoblot displaying E2F1, p53 and SDMe protein levels in WT E2F1 and E2F1 Cr p53^−/−^ HCT116 cells after 48 h of 1 μM PRMT5 inhibitor treatment (T1–44). **c** Venn diagrams showing the overlap of genes up- or downregulated with over 30% change (adjusted *P*-value threshold < 0.01) in each treatment condition with respect to DMSO-treated p53^−/−^ HCT116 cells, filtered for genes containing an E2F1 motif in their proximal promoter region (−900 to +100) based on ENCODE. T1–44, cells treated with PRMT5 inhibitor; E2F1Cr, E2F1 CRISPR cells; E2F1Cr T1–44, E2F1 CRISPR cells treated with PRMT5 inhibitor, *n* = 3 (see also Supplementary Data S1–S3). **d** Heatmap of differentially expressed E2F1 target genes (adjusted *P*-value ≤ 0.01, 30% change) in each treatment condition with respect to DMSO-treated p53^−/−^ HCT116 cells. 2Log fold-change expression values were converted to the *Z*-score. Increased expression levels are indicated with darker red colouring, whereas decreased expression levels are indicated with darker blue colouring. Ivory colour represents no significant change (see also Supplementary Data S1–S3). **e** Cluster analysis of genes co-expressed with cortactin/CTTN identified with weighted correlation network analysis (WGCNA) from triplicate experiments. Genes connected with focal adhesions are highlighted in red. WT E2F1, wild-type E2F1 p53^−/−^ HCT116 cells treated with DMSO; WT E2F1 T1–44, wild-type E2F1 p53^−/−^ HCT116 cells treated with T1–44.

We evaluated the effect of PRMT5 using a selective small molecule inhibitor, T1–44, which had, in a biochemical PRMT5 enzyme assay, an EC50 in the low nM range (Supplementary Fig. S1A). For comparison, we used a close chemical analogue, T1–68, which had reduced activity against PRMT5 (Supplementary Fig. S1A). Both compounds were tested side-by-side for effects in HCT116 cells in the MTT (3-(4,5-dimethylthiazol-2-yl)-2,5-diphenyltetrazolium bromide), colony formation and inhibition of the symmetrical arginine methyl-(R) mark assay, where T1–44 had activity in the low nM range and T1–68 exhibited about 200-fold reduced activity (Supplementary Fig. S1B-D). T1–44 demonstrated similar activity in both U2OS and MCF7 cells in colony formation and MTT assays (Supplementary Fig. S2B-E), whereas T47D cells appeared to be particularly responsive to T1–44, demonstrating around 20,000-fold increased sensitivity as compared with T1–68 (Supplementary Fig. S2F, G). Further, at 6 days of treatment of HCT116 cells, the most significant effect was an increased sub-G1 population with T1–44 where, in contrast, the effect of T1–68 was considerably reduced (Fig. 1a and Supplementary Fig. S1E).

Our recent studies identified E2F1 as a functionally relevant substrate for PRMT5^9,10^. To confirm this was the case in HCT116 cells, we prepared E2F1 knockout cells using CRISPR and assessed the effect of treating wild-type (WT E2F1) and CRISPR HCT116 cells (E2F1 Cr) with T1–44. We prepared the E2F1 knockout cells in both a p53^+/+^ and p53^−/−^ background to enable any influence of p53 to be assessed, given previous reports that p53 can be a target for PRMT5^12,13^. In a p53^−^^/−^ background, the sub-G1 population of cells was diminished in the E2F1 Cr compared with WT cells (25% reduction relative to WT cells; Fig. 1a and Supplementary Fig. S1F); in contrast, T1–68 treatment was not influenced by the status of E2F1 (Fig. 1a and Supplementary Fig. S1F) and the growth rate of E2F1 WT and Cr cells was very similar (Supplementary Fig. S1G). Furthermore, T1–44 treatment was less able to increase the sub-G1 population in the presence of p53 compared with the absence of p53 (p53^−/−^ cells; Fig. 1a and Supplementary Fig. S1E), and as in the presence of p53 there was minimal difference between E2F1 WT and Cr cells (Fig. 1a and Supplementary Fig. S1F), it appears that p53 influences the interplay between PRMT5 and E2F1. These results highlight the role of PRMT5 in maintaining tumour cell viability through E2F1 activity, which is most prevalent in p53-defective cells.

### Genome-wide effects of PRMT5 dependent on E2F1

We wanted to explore whether there are other roles for the PRMT5-E2F1 axis. To this end, we designed an RNA sequencing (RNA-seq) experiment to assess at the genome-wide level the influence of PRMT5 and E2F1, and mined the expression data for biologically relevant signatures. We performed the study in a p53^−/−^ background where the effect of PRMT5 inhibition and the role of E2F1 was most apparent (Fig. 1a). We treated either E2F1 WT or Cr HCT116 cells with PRMT5 inhibitor T1–44 (or the control treatment) and performed RNA-seq after 48 h under conditions of inhibition of the PRMT5 methyl-mark (Fig. 1b), as at this dose significant levels of sub-G1 cells were not observed (Supplementary Fig. S1E). There was also minimal effect in an MTT assay performed at this dose and time point (Supplementary Fig. S2A), thus reducing the influence of general cellular events on the RNA-seq data. We mined the RNA-seq data set for differentially expressed E2F target genes (DEGs) in each condition relative to the control treatment on WT cells, using a 30% or twofold change in expression level as the cut-off point with an adjusted *p*-value < 0.01 (Fig. 1c, d and Supplementary Fig. S3A, B). At the 30% cut-off, a large number of E2F target genes were seen to be DEGs, shown according to Venn and heatmap analysis (Fig. 1c, d). For example, in WT E2F1 cells treated with T1–44, a group of 216 genes were selectively upregulated dependent on E2F1, as they were absent in the E2F1 Cr cells, where a different set of 184 genes were upregulated. Interestingly, a set of 356 genes were upregulated in E2F1 Cr cells upon PRMT5 inhibition, highlighting a group of genes dependent on E2F1 and PRMT5 activity. A similar analysis was performed on downregulated DEGs where a unique set of genes (307) was apparent in the T1–44 treatment in WT E2F1-expressing cells (Fig. 1c), representing a group of genes under positive control by PRMT5 and dependent upon E2F1. A set of 141 genes exhibited reduced expression in E2F1 Cr cells and 367 genes were selectively downregulated upon inactivation of E2F1 and inhibition of PRMT5 (Fig. 1c). In general, these results define different sets of differentially expressed E2F target genes, representing genes regulated by E2F1 or PRMT5, and the third set regulated by both E2F1 and PRMT5.

We applied parametric gene set enrichment analyses (PGSEA)^14^ to test for enrichment of KEGG (Kyoto Encyclopedia of Genes and Genomes)-annotated pathways, GO vocabulary terms and highlight the differences between T1–44-treated and control samples. Some of the enriched GO and KEGG terms that were significantly different between T1–44 treatment and the control were connected with cellular adherence and migration, such as KEGG ‘Adherens Junction’ and KEGG ‘Leukocyte Transendothelial Migration’, and in the GO analysis ‘Filamentous actin’ was a highly ranked term (Supplementary Fig. S3C). Similar terms were also identified when comparing differential expression between control and E2F1 Cr samples, such as KEGG ‘Focal Adhesion’ and ‘Regulation of Actin Cytoskeleton’ terms, and in the GO analysis ‘Anchoring junction’ and ‘Actin Cytoskeleton’ terms (Supplementary Fig. S3D). We therefore interrogated the RNA-seq data for genes connected with the KEGG and GO terms, and identified a number as being highly regulated DEGs in the RNA-seq data sets, being generally downregulated upon PRMT5 inhibition (Fig. 1c). An example of one such gene was cortactin/CTTN, which ranked as one of the top DEGs after T1–44 treatment (Fig. 1c). We therefore performed co-expression analysis using cortactin/CTTN in the RNA-seq data by weighted correlation network analysis^15^, which identified a co-expression cluster containing several genes associated with focal adhesions (highlighted in red in Fig. 1e). Similar to cortactin/CTTN, genes within the co-expression cluster, including genes connected with focal adhesion, were generally downregulated upon PRMT5 inhibition (Fig. 1e).

**Fig. 2:**
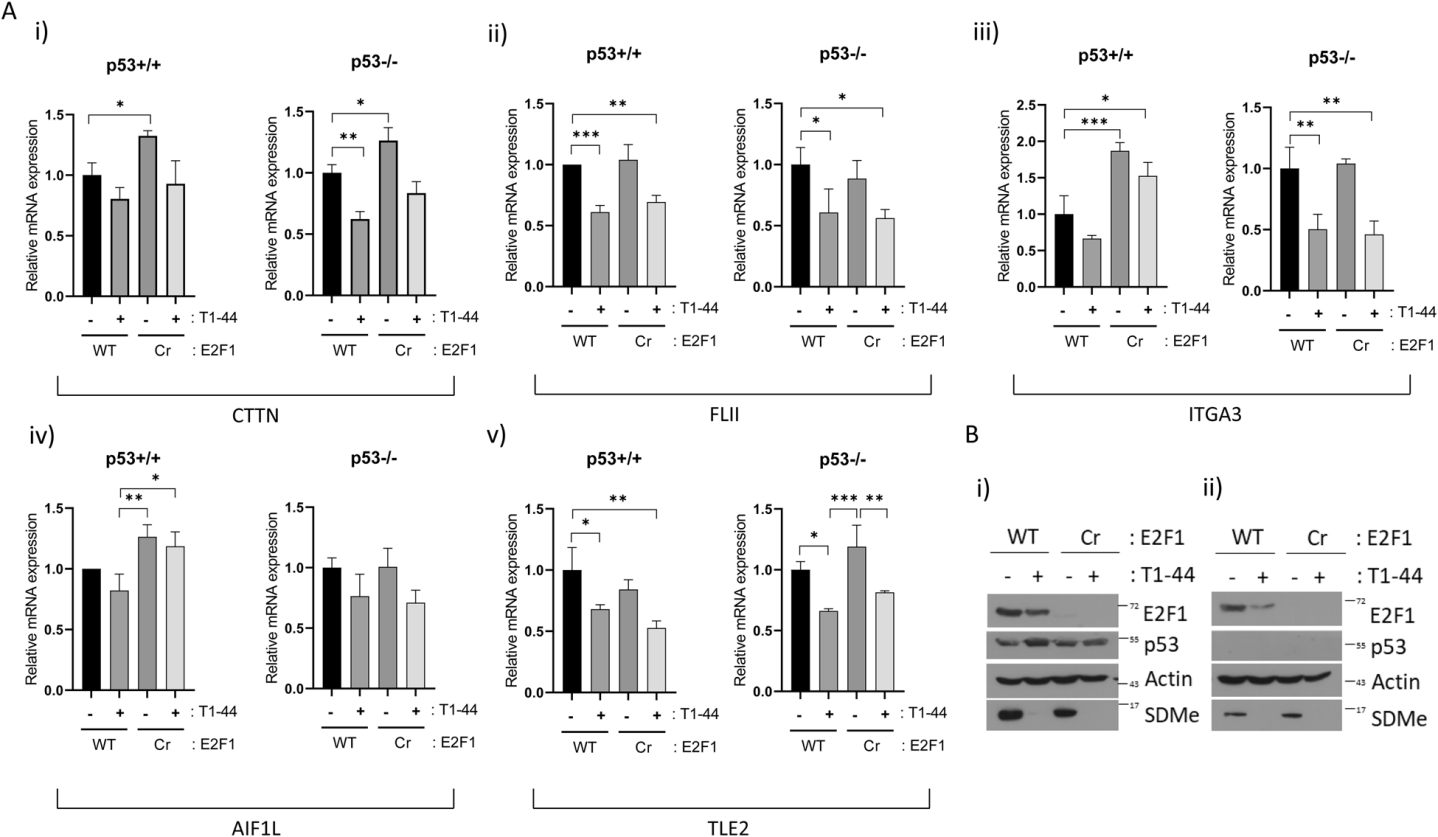
Downregulation of focal adhesion-related genes after PRMT5 inhibition. **a** Quantitative reverse-transcription PCR (qRT-PCR) of CTTN gene in WT E2F1 p53^+/+^ and p53^−/−^ HCT116 cell lines, and E2F1 Cr p53^−/−^ and p53^−/−^ HCT116 cells, treated for 2 days with 1 μM T1–44 or DMSO control (i); (ii) as above, but qRT-PCR was performed on the FLII gene; (iii) as above, but qRT-PCR was performed on the ITGA3 gene; (iv) as above, but qRT-PCR was performed on the AIF1L gene; (v) as above, but qRT-PCR was performed on the TLE2 gene. **b** An immunoblot of p53^+/+^ (i) and p53^−/−^ (ii) cells is included to demonstrate input protein levels for E2F1, p53 and SDMe; actin included as a loading control.

We further evaluated the expression of genes within this co-expression cluster by quantitative PCR (qPCR) in HCT116 cells under the same conditions as the RNA-seq analysis. The expression level of cortactin/CTTN, flightless I actin remodelling protein (FLII), integrin subunit-α3 (ITGA3), allograft inflammatory factor 1 like (AIF1L), and transducin-like enhancer protein 2 (TLE2) was reduced in cells treated with T1–44 compared with untreated cells, whereas loss of E2F1 also impacted on the expression of some of these genes (such as *cortactin*/*CTTN*, *ITGA3* and *AIF1L*) (Fig. 2a). In addition, the effect of T1–44 on gene expression was marginally influenced by p53 status (Fig. 2a). We also confirmed that these genes were a target for E2F1 by chromatin immunoprecipitation (ChIP) on their promoters (Supplementary Fig. S4, shown for cortactin/CTTN) or by analysing ChIP-sequencing (ChIP-seq) data for E2F1 from ENCODE project (https://www.encodeproject.org) (Supplementary Fig. S5, shown for cortactin/CTTN, FLII, AIF1L and ITGA3). E2F1 localized to the promoter of cortactin/CTTN in WT cells and was undetectable in E2F1 Cr cells (Supplementary Fig. S4). Furthermore, E2F1 ChIP activity was evident in both p53-expressing and p53^−/−^ cells (Supplementary Fig. S4).

### The PRMT5-E2F1 axis controls cell migration and invasion

**Fig. 3:**
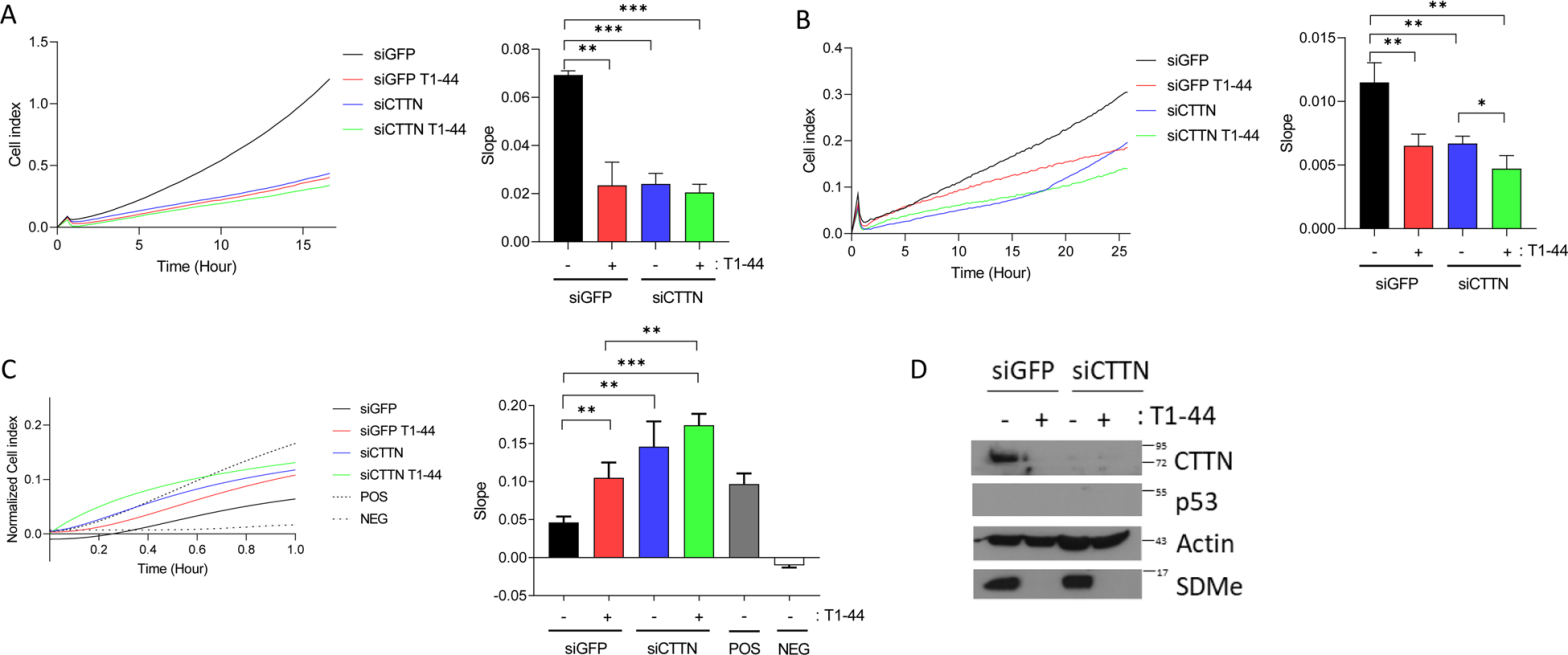
Cortactin/CTTN regulates motility through the PRMT5-E2F1 axis. **a** Migration assay performed in WT E2F1 p53^−/−^ HCT116 cells in the presence or absence of CTTN siRNA, after 2 days of treatment with 1 µM T1–44 or DMSO control. The rate of migration was determined by analysing the slope of the line between 4 and 10 h intervals, and is presented in the bar charts to the right. *n* = 3. **b** An invasion assay was performed under identical conditions as described above. The rate of invasion was determined by analysing the slope of the line between 4 and 8 h intervals, and is presented in the bar charts to the right *n* = 3. **c** An adhesion assay was performed under identical conditions as described above. The rate of adhesion was determined by analysing the slope of the line between 0 and 0.4 h intervals, and is presented in the bar charts to the right. *n* = 3. **d** An immunoblot demonstrating input protein levels for CTTN, p53 and SDMe for the experiments in **a**, **b** and **c** is displayed; actin included as a loading control.

The genes identified within the co-expression cluster have established biological functions in cell migration and invasion. For example, cortactin/CTTN itself is highly expressed in certain cancer types^16,17^ and plays a key role in cell motility as a cofactor for the branched actin nucleator complex Arp2/3^18^. It also binds a number of signalling and cytoskeletal proteins that mediate its role in cellular invasion^18^. The identification of focal adhesion/cell migration genes as E2F1 targets prompted us to assess the influence of the PRMT5-E2F1 axis on cell migration, invasion and adherence, which we measured in real-time by xCELLigence. To examine the relevance of genes such as cortactin/CTTN on adherence and migration, we additionally performed experiments with small interfering RNA (siRNA) treatment to reduce cortactin/CTTN expression levels in cells (Fig. 3).

Migration and invasion activity was reduced in T1–44-treated cells (Fig. 3a, b). This effect was mirrored by siRNA cortactin/CTTN treatment, when migration and invasion was reduced to a similar level to cells treated with T1–44 (Fig. 3a, b). The combined treatment of T1–44 and cortactin/CTTN siRNA did not result in any further significant reduction of migration (Fig. 3a), though a modest, yet statistically significant, reduction was observed in the invasion assay (Fig. 3b). In general, however, these results argue that PRMT5 and cortactin/CTTN are connected through a shared mechanism. It was also noteworthy that p53 did not significantly impact on the effect of T1–44 and cortactin/CTTN siRNA on migration and invasion activity (Supplementary Fig. S6A, B).

HCT116 cells treated with T1–44 underwent an increase in cell adhesion (Fig. 3c). Similarly, cortactin/CTTN depletion with siRNA caused an increase in adhesion (Fig. 3c). However, the combined treatment of T1–44 and cortactin/CTTN siRNA treatment had minimal difference from either treatment alone (Fig. 3c), again highlighting the possibility of a shared mechanism. The presence of WT p53 did not significantly impact the effect of T1–44 and cortactin/CTTN siRNA on adhesion (Supplementary Fig. S6C).

It is interesting to note that there was a reduction in cortactin/CTTN protein levels after T1–44 treatment (Fig. 3d and Supplementary Fig. S6D), which is consistent with the effect of T1–44 on cortactin/CTTN gene expression (Fig. 2a). This further argues in favour of a model in which PRMT5 and cortactin/CTTN are connected through a shared mechanism, as both siRNA knockdown of cortactin/CTTN or its downregulation via T1–44 treatment leads to the same expression pattern and physiological outcome.

**Fig. 4:**
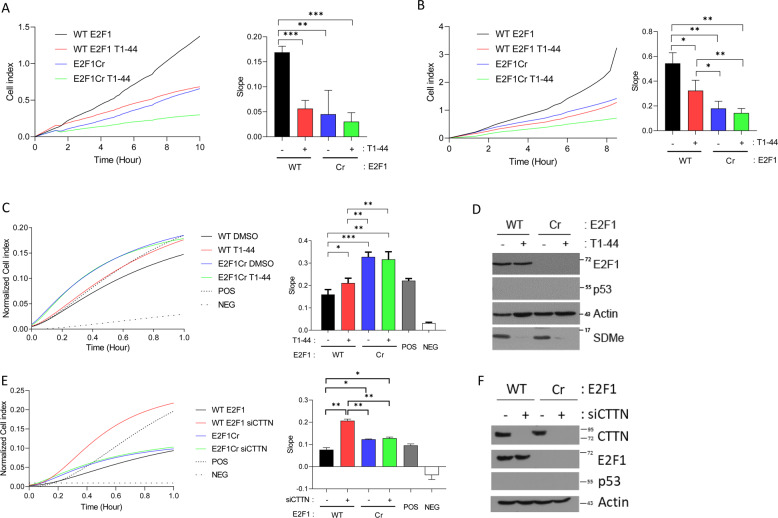
The PRMT5-E2F1 axis controls cell motility. **a** Migration assay performed in p53^−/−^ WT E2F1 and p53^−/−^ E2F1 Cr HCT116 cells after 2 days of treatment with 1 µM T1–44 or DMSO control. The rate of migration was determined by analysing the slope of the line between 4 and 10 h intervals, and is presented in the bar charts to the right. *n* = 3. **b** An invasion assay was performed under conditions identical to those described above. The rate of invasion was determined by analysing the slope of the line between 4 and 8 h intervals, and is presented in the bar charts to the right. *n* = 3. **c** An adhesion assay was performed under conditions identical to those described above. The rate of adhesion was determined by analysing the slope of the line between 0 and 0.4 h intervals, and is presented in the bar charts to the right. *n* = 3. **d** An immunoblot demonstrating input protein levels for E2F1, p53 and SDMe for the experiments in **a**, **b**, and **c** is displayed; actin included as a loading control. **e** Adhesion assay performed in p53^−/−^ WT E2F1 and p53^−/−^ E2F1 Cr HCT116 cells in the presence of CTTN siRNA. The rate of adhesion was determined by analysing the slope of the line between 0 and 0.5 h intervals, and is presented in the bar charts to the right. *n* = 3. **f** Immunoblot displaying input protein levels for CTTN, E2F1 and p53 for the experiment in **e** is presented; actin included as a loading control.

We then assessed the role of E2F1. E2F1 Cr cells exhibited defective migration and invasion activity compared to E2F1 WT cells (Fig. 4a, b). Treating E2F1 Cr cells with T1–44 did not result in a further reduction in migration and invasion (Fig. 4a, b). Inhibition of PRMT5 activity caused an increase on adhesion activity, as did loss of E2F1. Again, E2F1 Cr cells appeared unresponsive to PRMT5 inhibition since T1–44 treatment did not result in a further change to adhesion activity (Fig. 4c), suggesting that E2F1 is required for PRMT5 to influence adhesion. A similar effect of E2F1 loss and PRMT5 inhibition on adhesion activity occurred in cells expressing WT p53 (Supplementary Fig. S7C), though an additive effect of E2F1 loss and T1–44 treatment was observed for migration and invasion in p53^+/+^ cells (Supplementary Fig. S7A, B).

We related the effect of E2F1 on adhesion to cortactin/CTTN expression. As expected, cortactin/CTTN siRNA treatment caused an increase in cell adhesion in WT E2F1-expressing cells (Fig. 4e), but treating E2F1 Cr cells with cortactin/CTTN siRNA did not cause any additional change in adhesion activity (Fig. 4e), suggesting that cortactin/CTTN is a relevant target gene for E2F1 to influence adhesion. These results highlight a role for the PRMT5-E2F1 axis in regulating cell migration, invasion and cell adhesion.

### E2F1, PRMT5 and cortactin/CTTN expression in human cancer

**Fig. 5:**
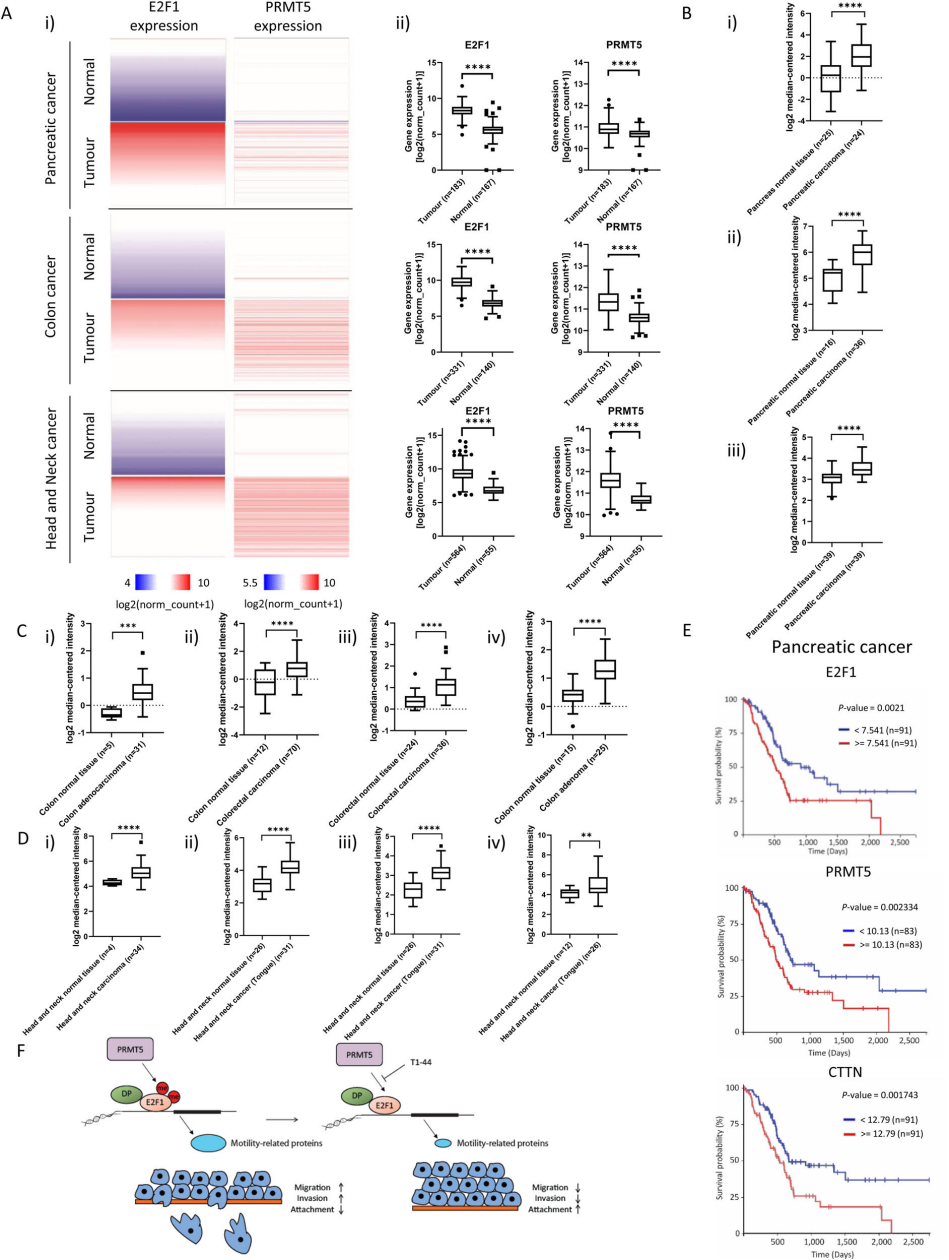
Regulation of E2F1, PRMT5 and cortactin/CTTN expression in human cancer. **a** Heatmap representation of expression levels for E2F1 and PRMT5 genes in human cancers (pancreatic, colon, and head and neck cancer) compared with normal tissue, generated using Xena Browser. Data from The Cancer Genome Atlas and Genotype-Tissue Expression projects were used to display expression levels from cancer tissue or healthy tissue, respectively. Each line on the heatmap represents a single patient sample and each column represents the expression level of a particular gene (E2F1, PRMT5). High expression levels are indicated with darker red colouring, whereas low expression levels are indicated with darker blue colouring (i); graphical representation of absolute expression values is also shown (ii). **b** Expression level of the cortactin/CTTN gene in different pancreatic cancer data sets (Ishikawa et al.^50^ (i), Pei et al.^51^ (ii) and Badea et al.^52^ (iii) data sets), generated using the Oncomine online tool, were plotted as log2 median intensity. **c** The expression level of the cortactin/CTTN gene in different colorectal cancer data sets (Kaiser et al.^46^ (i), Hong et al.^47^ (ii), Skrzypczak et al.^48^ (iii), and Sabates-Beliver et al.^49^ (iv) data sets), generated using the Oncomine online tool, were plotted as log2 median intensity. **d** The expression level of the cortactin/CTTN gene in different head and neck cancer data sets (Cromer et al.^53^ (i), Estilo et al.^54^ (ii), Talbot et al.^55^ (iii), and Ye et al.^56^ (iv) data sets), generated using the Oncomine online tool, were plotted as log2 median intensity. **e** Kaplan–Meier curves of overall survival of patients with pancreatic cancer for *E2F1*, *PRMT5* and *CTTN* genes are presented; generated using Xena Browser. For each analysis, patients were divided into two groups: one with high expression (above or equal to the mean gene expression of all patients; red line) or one with low expression (below the mean gene expression of all patients; blue line). **f** The model describes the interplay between PRMT5, E2F1 and motility regulating genes in migration, invasion and adhesion. By regulating the methylation of E2F1, it is proposed that PRMT5 can influence the expression level of motility-related genes. In the absence of E2F1 methylation (under conditions of PRMT5 inhibition), the expression of these genes is decreased, which results in a loss of cell motility.

Our results suggest that PRMT5 and E2F1 are linked through a shared pathway of control that impacts on the migration and invasion of cancer cells. To evaluate the expression of E2F1 and PRMT5 during human disease, we mined the TCGA database (The Cancer Genome Atlas Program; https://portal.gdc.cancer.gov/) and compared the expression profile of each gene to each other, in tumour and normal tissue. We observed coincidental high expression of E2F1 and PRMT5 in several tumour types, including pancreatic, colon, and head and neck cancer, with low expression in normal tissue (Fig. 5a). In all three cancer types, there was a similar high expression of cortactin/CTTN in tumour relative to normal tissue (Fig. 5b–d). By Kaplan–Meier analysis on pancreatic cancer, the level of each gene is linked to survival probability, with high expression for E2F1, PRMT5 and cortactin/CTTN linked to poor survival (Fig. 5e).

## Discussion

Cancer cells undergo certain fundamental changes, often termed ‘hall marks’, to attain the malignant phenotype. One of the key hall marks they acquire is self-sufficiency in growth signals and insensitivity to growth-inhibitory signals, which is in part mediated through deregulation of the pRb-E2F pathway^19,20^. The widespread if not universal deregulation of the pRb-E2F pathway underscores its importance in human cancer^19,20^.

Most tumours will acquire the ability to metastasize, beginning with invasion of the surrounding tissue from the site of the primary tumour^21^. Metastatic tumour cells are highly motile and invasive, which allows them to find a new tissue location to produce a secondary tumour^21^. It is recognized that metastatic disease is the primary cause of cancer morbidity and thus it is important to identify and understand mechanisms involved with tumour cell invasion and migration, which could in turn provide new targets for therapeutic intervention.

PRMT5 is an enzyme that is acquiring increased prominence in cancer^22–24^. It is expressed at high levels in diverse tumours where it has been suggested to promote invasion and migration^25,26^, observations consistent with the frequent up-regulation of PRMT5 expression in late stage disease^26–28^. Mechanistically, PRMT5 is a pleiotropic enzyme involved in diverse processes. It is, e.g., an established regulator of RNA biogenesis, in particular RNA splicing, which frequently becomes aberrant in cancer^24,29^. Further, PRMT5 is an upstream regulator of E2F1 activity^10^, where, in competition with PRMT1, it methylates discrete arginine residues on E2F1 that help direct its biological activity in to either proliferation or cell cycle arrest^10^.

The p53 tumour suppressor is another central player in cell cycle control which, like the pRb-E2F pathway, is under aberrant control in the majority of human tumours^30^. Activation of p53 largely occurs through protein stabilization, leading to a rapid increase in protein abundance and initiation of a p53 transcriptional response^30^. In previous studies, p53 was found to be a target for PRMT5, which in turn assisted the outcome of the p53 response^12,13^.

The results from our study shed light on the important role which E2F1 undertakes in mediating the cancer-relevant effects of PRMT5. By using E2F1 WT and Cr cells, combined with chemical inhibition of PRMT5 activity, we established unequivocally that E2F1 is crucial for PRMT5 to retain cancer cells in the proliferative state; thus, inactivating PRMT5 in E2F1 WT cells prompted increased levels of the sub-G1 fraction, highlighting a growth-promoting role for PRMT5 mediated by E2F1. Importantly, this was far more evident in cells with defective p53 activity, which, given the frequent inactivation of p53 in tumour cells, strengthens the cancer-relevance of the mechanism. It is possible that the influence of p53 on the biological consequences of PRMT5 activity is mechanistically related to the role of PRMT5 in the p53 response^12,13^.

Our results provide additional and strong evidence that PRMT5 promotes cancer cell invasion and migration. Through a genome-wide RNA-seq analysis of the PRMT5-E2F1 axis,* we identified a set of E2F target genes enriched with GO descriptors connected with cellular adherence. The gene set included cortactin/CTTN and other functionally related genes such as *FLII*, *TLE2*, *ITGA3* and *AIF1L*.

*Cortactin*/*CTTN* is a gene of significant interest in cancer cell invasion and migration because of its role as a crucial regulator of actin cytoskeletal dynamics and it’s overexpression in aggressive cancers^16,17^. Cortactin/CTTN binds to filamentous actin and functions as a nucleation-promoting factor that activates the Arp2/3 complex to facilitate actin polymerization and cell motility^16,31^. Overexpression of cortactin/CTTN is linked to metastatic disease in head and neck cancer, breast cancer, oesophageal cancer, hepatocellular carcinoma, melanoma and colorectal cancer^32–39^. Indeed, our studies support this conjecture as regulating cortactin/CTTN levels in vitro had a direct impact on invasion and migration of cancer cells.

Most significantly, our results connect PRMT5 activity and its regulation of E2F1 with the control of migration and invasion (Fig. 5F). This is important as it provides a mechanism which expands the influence of the pRb-E2F pathway from its well established role in cell cycle progression, to encompass cell migration and invasion; specifically, tumour cells which harbour defective pRb and enhanced E2F1 activity through PRMT5 would, in addition to the impact on proliferation, exhibit enhanced invasive properties by virtue of increased expression of genes such as *cortactin*/*CTTN*.

An analysis of PRMT5 and E2F1 expression suggests that the relationship, we have established has clinical relevance, as in diverse types of human cancer high level expression in tumour tissue was apparent, and within some cancers a further coincidence was apparent between the expression of PRMT5, E2F1 and cortactin/CTTN. This was particularly evident in pancreatic cancer, where the expression level of each gene was also associated with poor prognosis. In colorectal and head and neck cancer, a similar coincidence was apparent between the high expression of each gene in cancer tissue and the low expression in normal tissue.

In conclusion, our results show for the first time that the PRMT5-E2F1 axis not only influences cancer cell growth and division, but in addition augments the migration and invasive properties of tumour cells. As a positive regulator of the E2F1 axis, this information endorses PRMT5 as a viable therapeutic target and further suggests drugs targeting PRMT5 will find clinical utility in metastatic disease.

## Material and methods

### Cell line generation, culture and compound treatments

WT p53 and p53^−/−^ HCT116 E2F1 CRISPR cells were generated from cell lines (ATCC) according to the protocol described by Ran et al.^40^. E2F1 CRISPR single guide RNA sequence: 5′-GCATTCTTCTTCTGGCTGGG-3′. HCT116, MCF7, U2OS and T47D (ATCC) cells were cultured in Dulbecco’s modified Eagle medium (DMEM) (Sigma-Aldrich, St. Louis, MO, USA) supplemented with 10% fetal bovine serum (Labtech, Heathfield, UK) and 1% penicillin/streptomycin (Gibco, Life Technologies, Carlsbad, CA, USA). All cell lines were tested for mycoplasma contamination before use. Selective PRMT5 inhibitor (T1–44) and the less active analogue (T1–68) (synthesized by Argonaut Therapeutics Ltd, Oxford, UK) (Supplementary Table S1) were used for 48 h at 1 μM final concentration, unless otherwise stated. T1–44 is a close derivative of EPZ015666^41^ and exhibits high specificity for PRMT5 (EC50 of T1–44 against asymmetric PRMT1 enzyme was 1.88 mM).

### siRNA transfection

RNA interference was performed with 25 nM siRNA for 72 h using the Oligofectamine transfection reagent (Invitrogen, Carlsbad, CA, USA), as per the manufacturer’s instructions. Sequences for siRNA are as follows: nontargeting control: 5′-AGCUGACCCUGAAGUUCUU-3′, esiRNA human CTTN was provided by Sigma-Aldrich (EHU093121).

### MTT assay

MTT assay was performed as previously described^42^. Briefly, 1000 cells per well were seeded in 96-well plates. On the next day, appropriate treatments were administrated (in triplicate) and cells were cultured for 1–6 days. MTT activity was measured by treating cells with 10 μl MTT reagent (Sigma-Aldrich) per well and incubating at 37 °C for 2 h. The medium was removed and 100 μl dimethyl sulfoxide (DMSO) was added to each well and incubated at room temperature for 10 min with agitation. Absorbance was measured at 570 nm with a reference reading at 670 nm.

### PRMT5 activity assay

PRMT5 enzyme activity was measured using the PRMT5 Chemiluminescent Assay Kit (AMS Biotechnology, Abingdon, UK) according to the manufacturer’s protocol. Briefly, *S*-adenosylmethionine was incubated with a sample containing assay buffer and methyltransferase enzyme for 1 h. Next, primary antibody was added. Finally, the plate was treated with an horseradish peroxidase (HRP)-labelled secondary antibody followed by addition of the HRP substrate to produce chemiluminescence, which was measured by a FLUOstar Omega reader (BMG Labtech, Ortenberg, Germany).

### Cell proliferation

Cells were seeded at a density of 50,000 cells in triplicate (day 1). Cell counts were performed 2–6 days post seeding using a Luna-II™ Automated Cell Counter (Logos Biosystems, Villeneuve d’Ascq, France).

### Colony formation assay

Cells were seeded at a density of 1000 cells per well in a 6-well plate and were incubated with the indicated compounds for 8 days as described previously^10^. The culturing media was gently aspirated and the plates were briefly rinsed with cold phosphate-buffered saline (PBS). Crystal violet stain was applied to the cells for 2 min, followed by washing with deionized water and left to dry. Image scanning was performed with a Gelcount automated colony counter (Oxford Optronics, Oxford, UK).

### Immunoblots and antibodies

For immunoblottings, cells were collected in radio-immunoprecipitation assay buffer (50 mM tris-HCl (pH 8), 150 mM NaCl, 1% Igepal CA-630, 0.5% sodium deoxycholate, 0.1% SDS, 0.2 mM sodium orthovanadate and protease inhibitor cocktails) and incubated on ice for 30 min prior to SDS-polyacrylamide gel electrophoresis and transferred to nitrocellulose. The following antibodies were used in immunoblottings: β-actin (AC-74, Sigma-Aldrich), E2F1 (C20, Santa Cruz Biotechnology, SC-193, Dallas, TX, USA), E2F1 (Cell Signaling, 3742S, Danvers, MA, USA), p53 (DO-1, Santa Cruz Biotechnology, SC-126), CTTN (H222, Cell Signaling, 3503S) and SDMe (Cell Signaling, 13222S).

### RNA isolation and qPCR

RNA was isolated from cells using TRIzol (Thermo Fisher Scientific, Waltham, MA, USA) or the Direct-zol RNA MiniPrep kit (Zymo Research, Irvine, CA, USA) according to the manufacturer’s instructions. One microgram of total RNA was used for complementary DNA synthesis. Reverse transcription with oligo(dT)20 primer (Invitrogen) was performed using SuperScript III Reverse Transcriptase (Invitrogen) as per the manufacturer’s instructions. Quantitative reverse-transcription PCR was carried out in technical triplicate using the indicated primer pairs and the Brilliant III Ultra-Fast SYBR® Green QPCR Master Mix (Agilent, Santa Clara, CA, USA) on an AriaMX real-time qPCR instrument (Agilent). Results were expressed as average (mean) fold change compared with control treatments using the ΔΔCt method from three biological repeat experiments. Glyceraldehyde-phosphate dehydrogenase primer sets were used as an internal calibrator. Error bars represent SE unless otherwise indicated. Primers sets used for qPCR: CTTN forward: 5′-GATAAGTCAGCTGTCGGCCA-3′, CTTN reverse: 5′-ACACCAAACTTGCCTCCGAA-3′; FLII forward: 5′-ACCAGGATGTATCGTGTGTATGG-3′, FLII reverse: 5′-TCCAGAGAGGTCCCCTTGAG-3′; AIF1L forward: 5′-AGAACCTTCCAGAAAAGCTCACA-3′, AIF1L reverse: 5′-TCGCCTTCATTGTTCAGGTCA-3′; TLE2 forward: 5′-GGCTCAATACCACAGCCTCA-3′, TLE2 reverse: 5′-ATGTCGCTGCATTTCCGTCT-3′; ITGA3 forward: 5′-GCGCAAGGAGTGGGACTTAT-3′, ITGA3 reverse: 5′-CTGCATCGTGTACCCAATATAGA-3′.

### Chromatin immunoprecipitation

ChIP analysis was performed as described previously^42^. Antibodies used for immunoprecipitation were as follows: anti-E2F1 (A300-766A, Bethyl Laboratories, Montgomery, TX, USA) and nonspecific rabbit or mouse immunoglobulin G (IgG). The recovered DNA was analysed in technical triplicate by qPCR, as described^43^, on an AriaMX real-time qPCR system using the Brilliant III Ultra-Fast SYBR® Green QPCR Master Mix according to the manufacturer’s instructions. The percentage enrichment over input was calculated for both the E2F1 ChIP and the IgG controls from triplicate biological repeat samples. The data were then presented as average (mean) fold change compared to IgG control treatment. Error bars represent SE, unless otherwise indicated. ChIP primers: CTTN forward: 5′-AGAGATGAAGAGGCTCCCCG-3′, CTTN reverse: 5′-AGAGCTCGCCCGGAAGTA-3′; CDC6 forward: 5′-GGCCTCACAGCGACTCTAAGA-3′, CDC6 reverse: 5′-CTCGGACTCACCACAAGC-3′; Actin forward: 5′-ATCGTGCGTGACATTAAGGAGAAG-3′, Actin reverse: 5′-CTGGAAGCAGCCGTGGCCATCTCTTG-3′.

### Flow cytometry

Cells were treated with PRMT5 inhibitor T1–44 or the less active analogue T1–68 for 1–6 days as indicated. Next, cells were fixed in ice cold 70% ethanol/PBS and cell cycle analysis was performed using propidium iodide staining as previously described^44^. Samples were analysed with a BD Accuri™ C6 Plus Flow Cytometer (Becton Dickinson, Franklin Lakes, NJ, USA).

### Adhesion, migration and invasion assays

Cells (100,000 cells) were seeded in 6 cm dishes and treated with 1 µM PRMT5 inhibitor for 48 h. Adhesion, migration and invasion assays were carried out according to the manufacturer’s protocol using E-plates 16 and CIM plates 16 (Acea Biosciences, San Diego, CA, USA), respectively. All measurements were performed using an xCELLigence® RTCA DP Instrument. Briefly, for an adhesion assay Matrigel (prepared according to manufacturer’s instructions by mixing with DMEM medium in a 1 : 1 ratio; concentration 50%) (Cell Applications, 126-2.5, San Diego, CA, USA) was added to the wells in the E-Plate and incubated for 3 h at 37 °C. The coated plates were washed with PBS and incubated with 0.1% bovine serum albumin for 30 min at 37 °C. After PBS washing, 100 µl of media without fetal bovine serum (FBS) (serum free) was added to each well and the background impedance was measured. Cells were collected by short trypsinization, centrifuged and re-suspended in serum-free media. Cells (25,000 cells) suspended in 100 µl of media were transferred to Matrigel-coated wells. As a positive control, we used non-treated cells suspended in FBS-containing medium. As a negative control, empty Matrigel-coated wells without cells were used. The measurement of cell adhesion was monitored every 10 s for 1 h.

For an invasion assay, the upper chamber of a CIM plate was coated with Matrigel and incubated for 3 h at 37 °C. Then, a CIM-Plate 16 was assembled (lower chamber contains media with FBS; upper chamber contains serum-free media), equilibrated at 37 °C for 1 h and the background measurement was taken. Preparation of cells was as described for the adhesion assay, although CIM plates were left for a 30 min to allow cells to settle. The measurement of cell invasion was monitored every 10 min for 72 h.

An identical protocol was used for the migration assay, but without the Matrigel coating step. The assay system expresses impedance in arbitrary Cell Index units (*R*_n_ − *R*_b_)/4.6; where *R*_n_ is the cell-electrode impedance of the well when it contains cells and *R*_b_ is the background impedance of the well with the media alone. The rate of adhesion/migration/invasion was determined by analysing the slope of the line between two given time points in each biological repeat (*n* = 3).

### RNA sequencing

WT E2F1 and E2F1 Cr p53^−/−^ HCT116 cells were treated for 48 h with 1 µM concentration of PRMT5 inhibitor (T1–44) or DMSO as a negative control. Total RNA from WT E2F1, WT E2F1 T1–44, E2F1 Cr and E2F1 Cr T1–44 (triplicates) was isolated using Direct-zol RNA MiniPrep kit (Zymo Research) according to the manufacturer’s instructions. RNA-seq was performed by BGI Genomics. Briefly, an Agilent 2100 Bioanalyzer (Agilent RNA 6000 Nano Kit) was used for RNA sample quality control purposes (RNA concentration, RNA integrity number (RIN) value, 28S/18S and the fragment length distribution). mRNAs were isolated from total RNA using the oligo(dT) method. Then the mRNAs were fragmented and first-strand/second-strand cDNA were synthesized. cDNA fragments were purified and resolved with EB buffer for end reparation and single-nucleotide A (adenine) addition. Subsequently, the cDNA fragments were linked with adapters. Those cDNA fragments with suitable size were selected for the PCR amplification. Agilent 2100 Bioanalyzer and ABI StepOnePlus Real-Time PCR System were used in quantification and qualification of those libraries. The RNA-seq was carried out using Illumina HiSeq Platform and 5.12 Gb per sample was generated.

### RNA-seq analysis

FASTQ files for p53^−/−^ WT E2F1 and p53^−/−^ E2F1 Cr HCT116 cells, treated with PRMT5 inhibitor or DMSO control were generated from three biological repeat experiments. These were trimmed to remove adapters and low-quality bases with TrimGalore v.0.4.3 (http://www.bioinformatics.babraham.ac.uk/projects/trim_galore/). The trimmed reads were aligned to the human reference genome build hg19 with STAR aligner v.2.7 with two mismatches allowed. Differential gene expression analysis was performed with DESeq2 R Bioconductor package (v.1.25.17), using read counts data provided by the aligner. Genes were considered differentially expressed if the adjusted *P*-value, calculated using the Benjamini–Hochberg method in order to minimize the false discovery rate, was less than 0.01. We further filtered significantly DEG sets using a 30% or twofold change in absolute expression level. RNA-seq data sets have been deposited to the Gene Expression Omnibus under accession code GSE142430.

### Parametric GSEA

The PGSEA was performed with the R PGSEA package (v. 1.58) on the collections of curated gene sets (c2, c5) derived from the KEGG pathway database and GO vocabulary, respectively, available from the Broad Institute’s Molecular Signatures Database (v 6.2). The expression matrix used in these analyses was normalized and rlog-transformed with DESeq2 R package. Gene sets with less than 10 genes and more than 10,000 genes were excluded from the analyses. A linear model was applied employing the limma package (v.3.44.0)^45^ followed by empirical Bayesian analysis to determine concepts associated with significant differences between treated and untreated samples. Differences were considered significant if the adjusted *P*-value, calculated using the Benjamini–Hochberg method, to minimize false discovery rate, was <0.005.

### Xena browser functional genomics analysis

For the analysis of E2F1, PRMT5 and CTTN expression levels in human cancers, Xena browser (University of California) was used (https://xena.ucsc.edu/). The TCGA TARGET GTEx data set was selected, which contained transcript expression data from TCGA (cancer tissue) and Genotype-Tissue Expression (GTEx; healthy tissue) samples. For subsequent detailed analysis of survival, phenotypic characteristics and correlation, pancreatic, colon, and head and neck cancer data sets from TCGA were used. Box plots show the inter-quartile range (box) and median (solid line). The whiskers show lowest and highest datum still within 1.5× inter-quartile range of the lower and upper quartiles. Outliers are shown as black dots.

### Oncomine analysis

Using the Oncomine online tool (www.oncomine.org), expression levels of the cortactin/CTTN gene were viewed in different colorectal, pancreatic, and head and neck cancer data sets, and compared against patient’s normal tissue. For colon cancer, the Kaiser et al.^46^, Hong et al.^47^, Skrzypczak et al.^48^ and Sabates-Beliver et al.^49^ data sets were analysed. For pancreatic cancer the Ishikawa et al.^50^, Pei et al.^51^ and Badea et al.^52^ data sets were analysed. For head and neck cancers the Cromer et al.^53^, Estilo et al.^54^, Talbot et al.^55^, and Ye et al.^56^ data sets were analysed. Expression data were presented as log2 median intensity and plotted using Graphpad Prism software. Box plots show the inter-quartile range (box) and median (solid line). The whiskers show lowest and highest datum still within 1.5× inter-quartile range of the lower and upper quartiles. Outliers are shown as black dots.

### ENCODE ChIP-seq data analysis

Cortactin/CTTN, FLII, AIF1L and ITGA3 promoter characterization was performed utilizing bioinformatics tools present in UCSC Genome Browser (https://genome.ucsc.edu; GRCh37/h19 assembly) and analysing ChIP-seq data for E2F1 tracks from the ENCODE project (http://genome.ucsc.edu/ENCODE/). The ‘Txn factor ChIP track’, ‘ENCODE 3 TFBS Track’, ‘Uniform TFBS track’ and ‘SYDH TFBS track’ tools were used to display E2F1 ChIP-seq peaks or signal as appropriate.

### Statistical analysis

Statistical analyses were performed using two-tailed, unpaired Student’s *t*-test and one-way analysis of variance test with GraphPad Prism 8 Software (GraphPad Software). Data are shown as means with SE displayed, unless otherwise indicated. *P*-values < 0.05 were considered significant and are labelled by *p < 0.05, **p < 0.01, ***p < 0.001, and ****p ≤ 0.0001.

## Supplementary information

Supplementary Figure

Supplementary Figure

Supplementary Figure

Supplementary Figure

Supplementary Figure

Supplementary Figure

Supplementary Figure

Supplementary Figures and Table legends

Supplementary Table S1

Supplementary Data 1

Supplementary Data 2

Supplementary Data 3
