# Supplementary figures and images for: PRMT5 promotes cancer cell migration and invasion through the E2F pathway
Source: Cell Death Dis. 2020 Jul 24;11(7):572. doi: 10.1038/s41419-020-02771-9 (PMC7382496; doi:10.1038/s41419-020-02771-9)

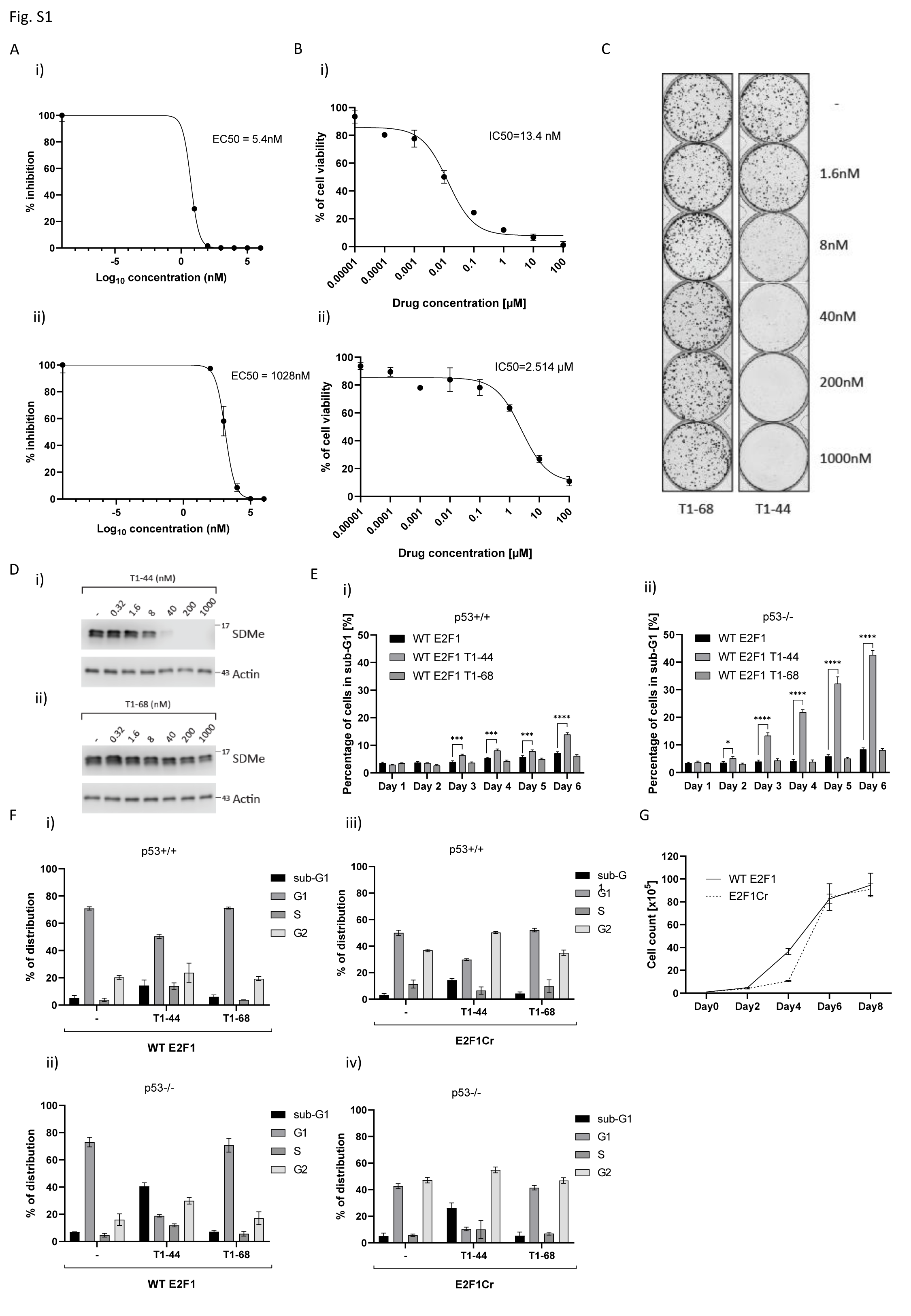

Supplement: Supplementary file 1 — Supplementary Figure [file 41419_2020_2771_MOESM1_ESM.tif]

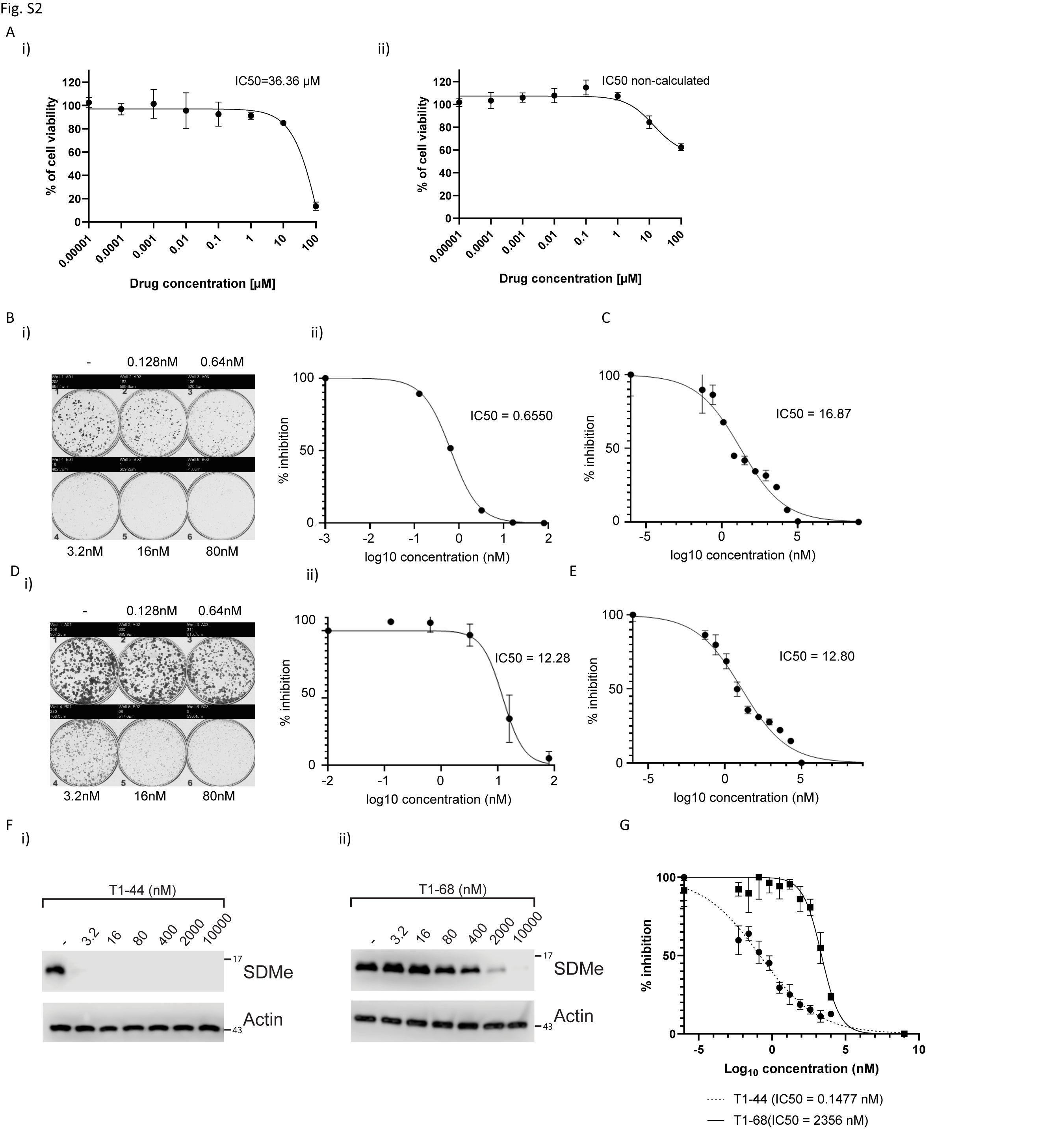

Supplement: Supplementary file 2 — Supplementary Figure [file 41419_2020_2771_MOESM2_ESM.tif]

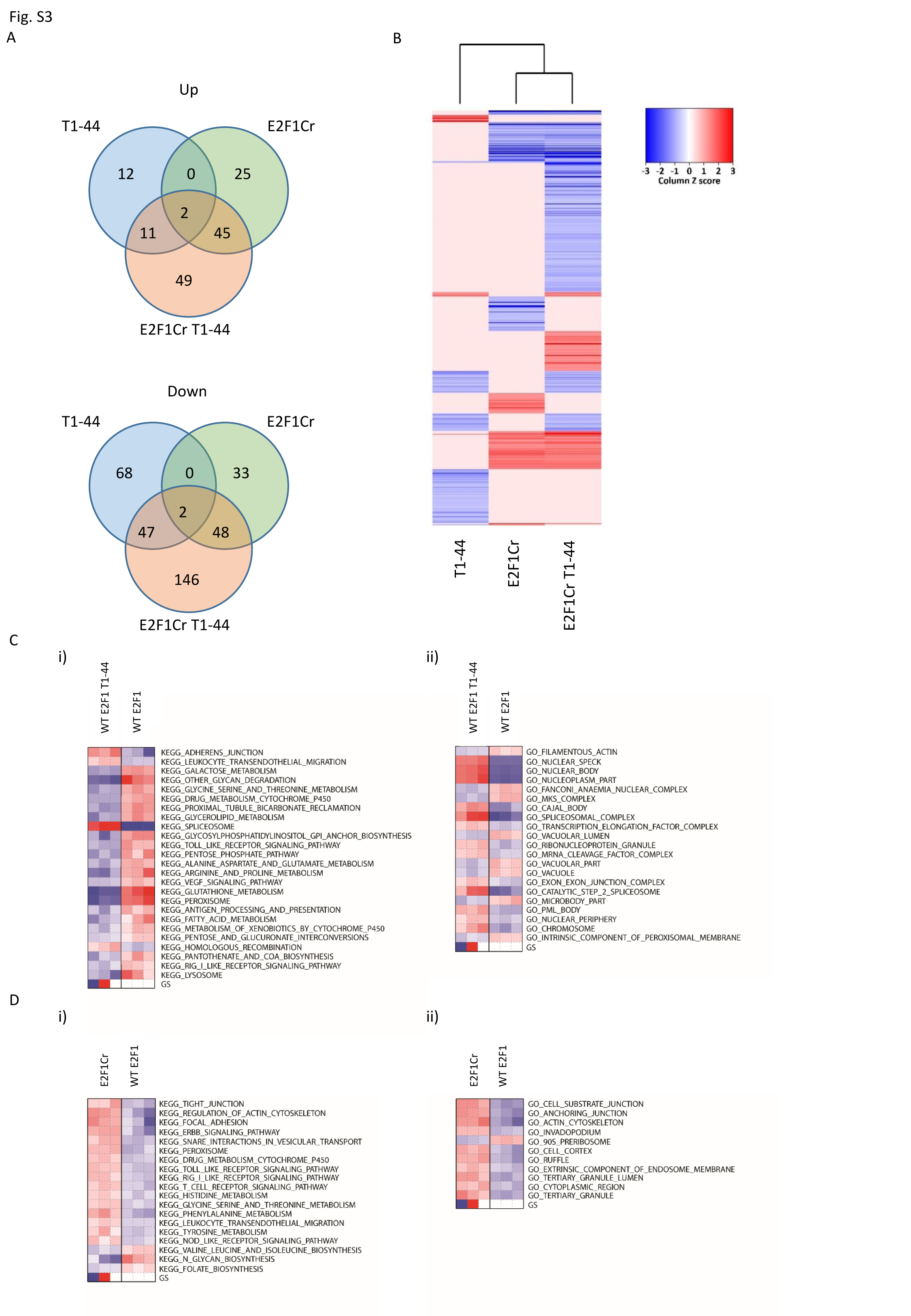

Supplement: Supplementary file 3 — Supplementary Figure [file 41419_2020_2771_MOESM3_ESM.tif]

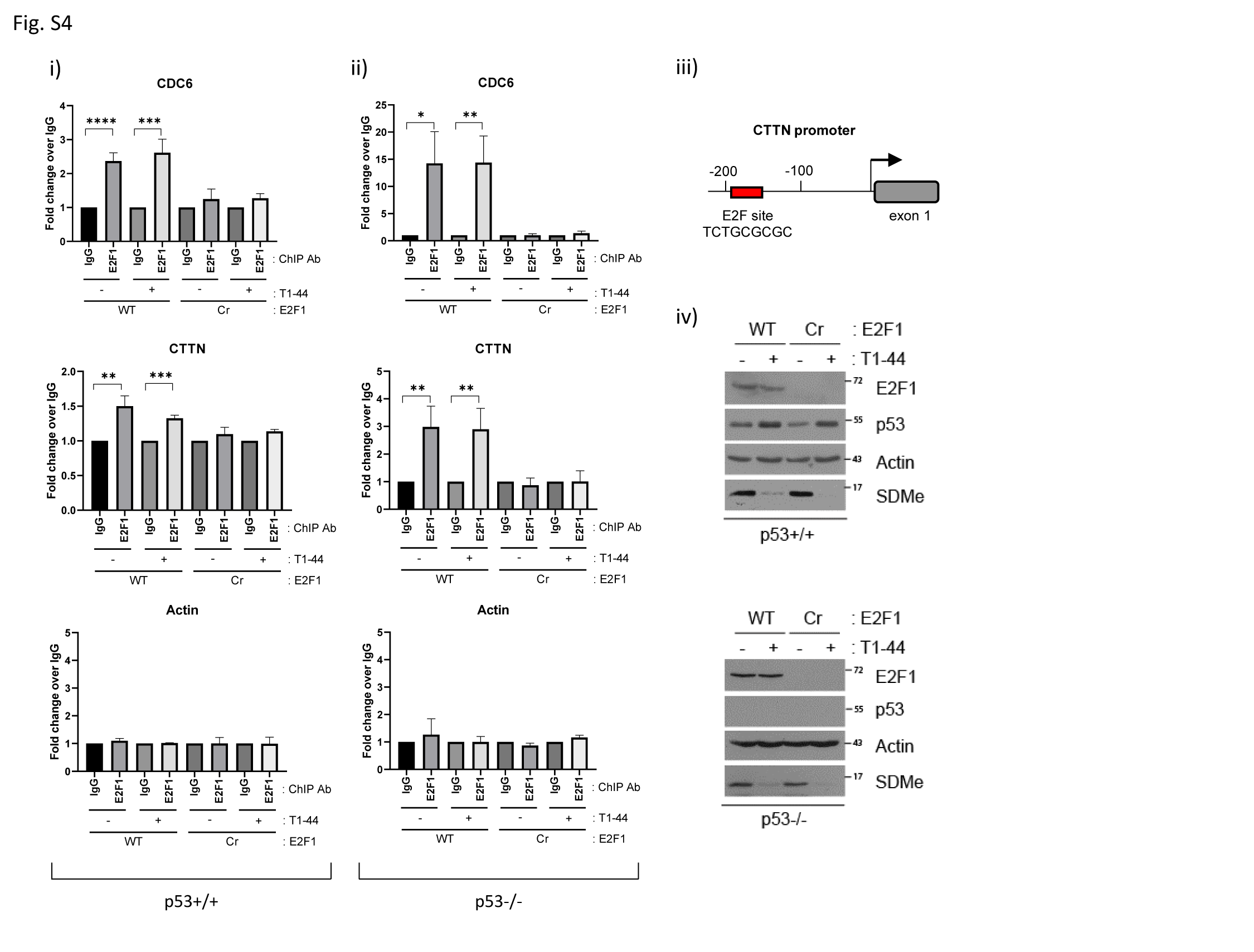

Supplement: Supplementary file 4 — Supplementary Figure [file 41419_2020_2771_MOESM4_ESM.tif]

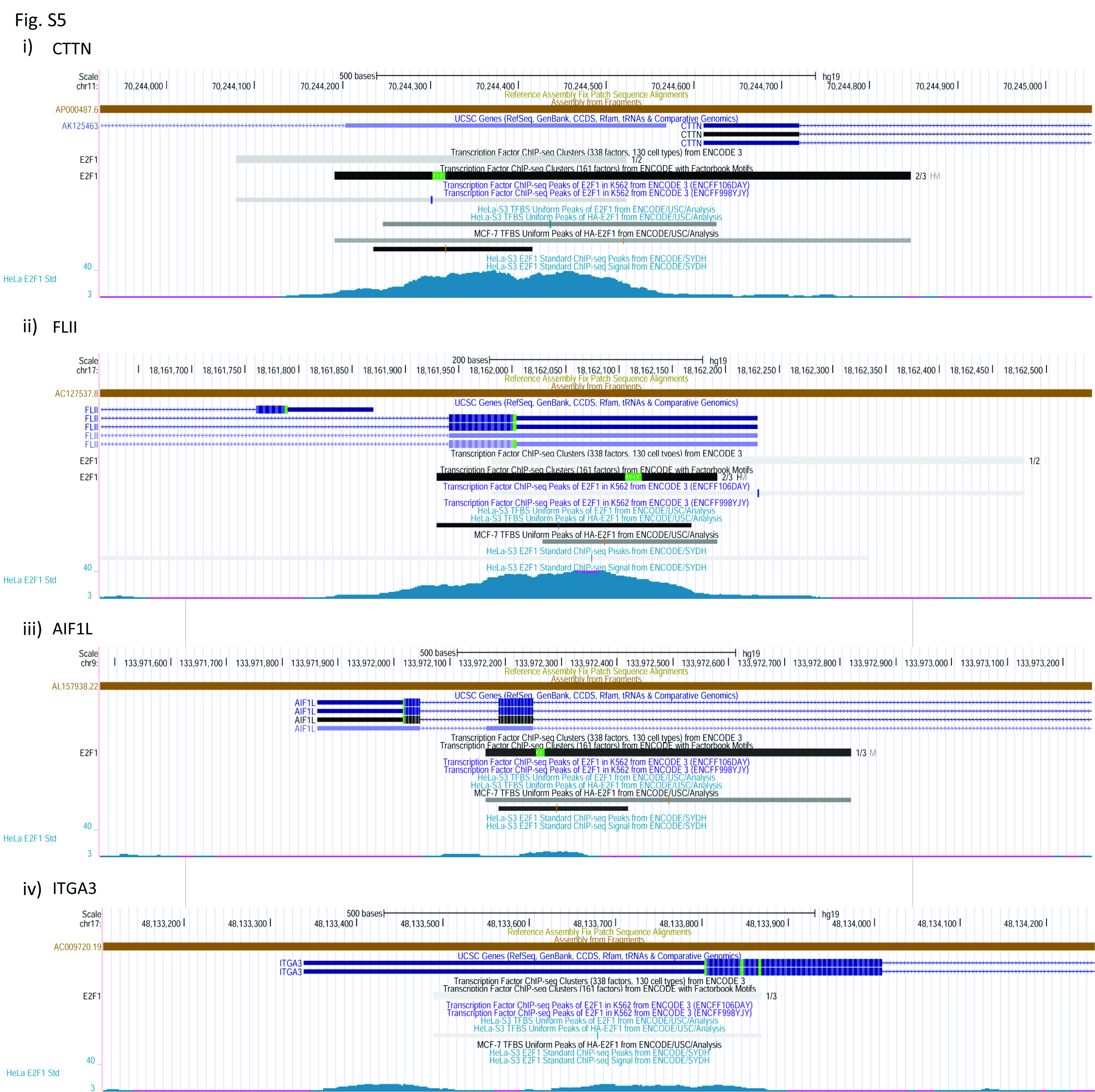

Supplement: Supplementary file 5 — Supplementary Figure [file 41419_2020_2771_MOESM5_ESM.tif]

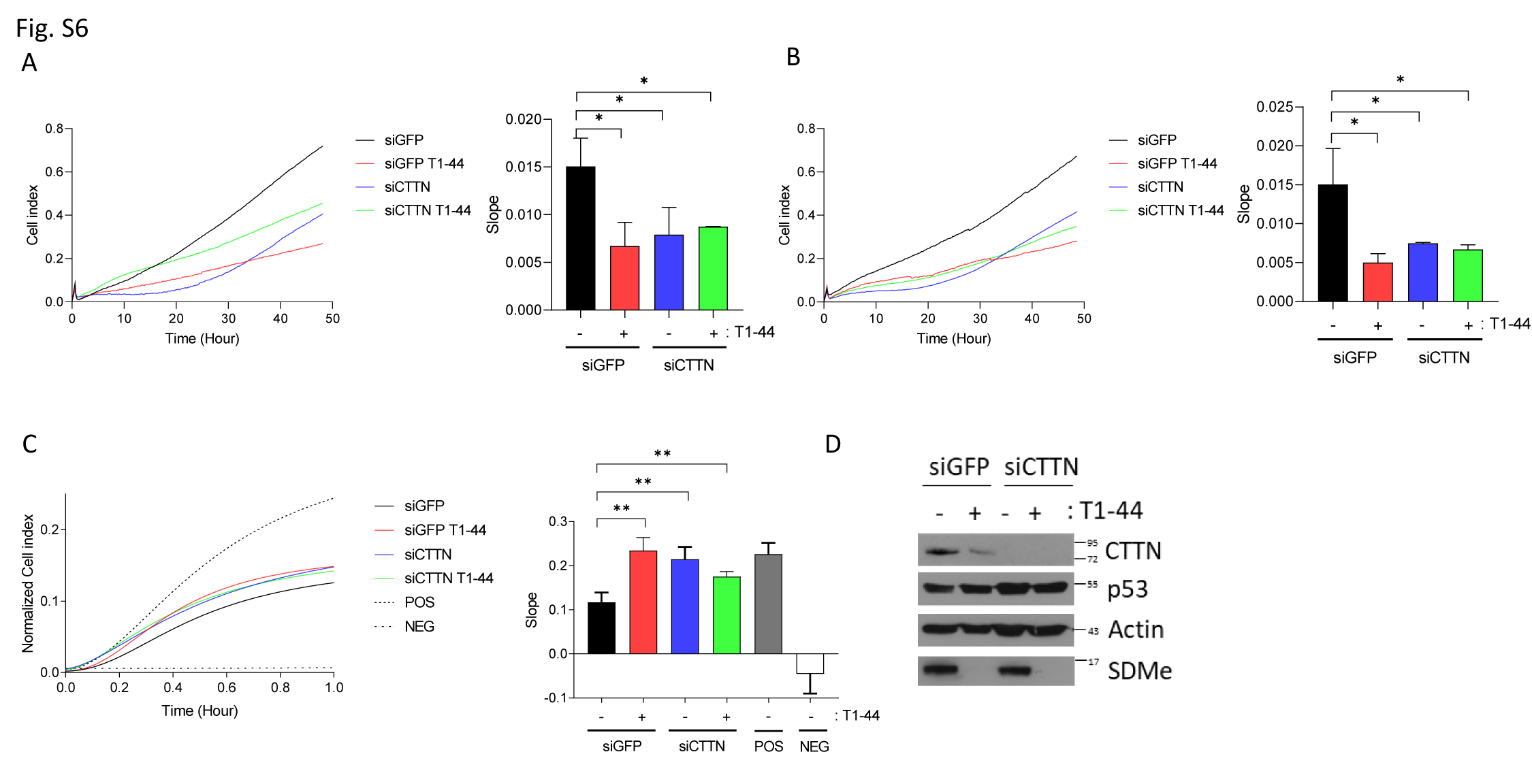

Supplement: Supplementary file 6 — Supplementary Figure [file 41419_2020_2771_MOESM6_ESM.tif]

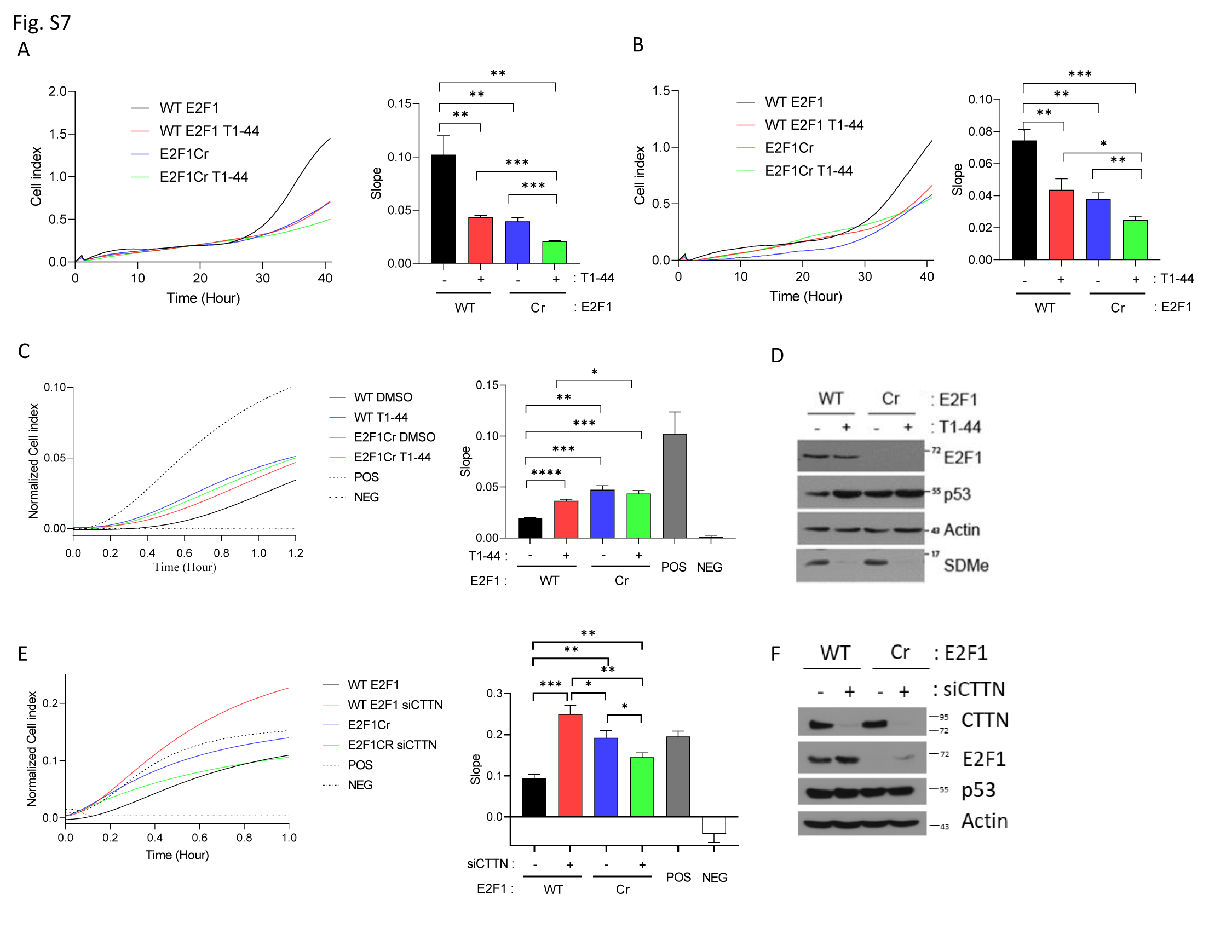

Supplement: Supplementary file 7 — Supplementary Figure [file 41419_2020_2771_MOESM7_ESM.tif]
