# Supplementary material for: PRMT5 promotes cancer cell migration and invasion through the E2F pathway
Source: Cell Death Dis. 2020 Jul 24;11(7):572. doi: 10.1038/s41419-020-02771-9 (PMC7382496; doi:10.1038/s41419-020-02771-9)
Supplement: Supplementary file 8 — Supplementary Figures and Table legends [file 41419_2020_2771_MOESM8_ESM.docx]

Supplementary figure and table legends for “PRMT5 promotes cancer cell migration and invasion through the E2F pathway”

Wojciech Barczak, Li Jin, Simon Mark Carr, Shonagh Munro, Samuel Ward, Alexander Kanapin , Anastasia Samsonova, Nicholas B La Thangue*

Nicholas B La Thangue

Email: nick.lathangue@oncology.ox.ac.uk

Fig. S1. Effect of PRMT5 inhibition is influenced by E2F1 status. A) EC50 plots of recombinant PRMT5 enzyme activity tested in an in vitro assay after treatment with increasing concentrations of T1-44 (i) and its less active analogue T1-68 (ii), n = 3, B) IC50 plots from MTT assays performed after treating HCT116 cells for 6 days with increasing concentrations of T1-44 (i) and T1-68 (ii). C) A colony formation assay in HCT116 cells was performed for 8 days after treatment with increasing concentrations of T1-44 and T1-68 (T1-44 IC50 = 5.15 nM), D) An immunoblot demonstrating SDMe levels in HCT116 cells after 6 days of treatment with increasing concentrations of T1-44 (i) and T1-68 (ii); actin included as a loading control. E) sub-G1 fraction analysis of WT E2F1 p53+/+ (i) and WT E2F1 p53-/- (ii) HCT116 cells after 6 days of treatment with 1 μM T1-44 or T1-68, n = 3. F) Cell cycle analysis of WT E2F1 p53+/+ (i), WT E2F1 p53-/- (ii), E2F1 Cr p53+/+ (iii) and E2F1 Cr p53-/- (iv) HCT116 cells after 6 days of treatment with 1 μM T1-44 or T1-68, n = 3. G) Proliferation analysis of p53-/- WT E2F1 and p53-/- E2F1 Cr HCT116 cells, n = 3.

Fig. S2. Effect of PRMT5 inhibition is influenced by E2F1 status. A) IC50 plots from MTT assays performed after treating HCT116 cells for 2 days with increasing concentrations of T1-44 (i) and T1-68 (ii). B) A colony formation assay in MCF7 (p53+/+) cells was performed for 8 days after treatment with increasing concentrations of T1-44 (i); corresponding IC50 plot is presented (ii). C) IC50 plot from MTT assays performed after treating MCF7 (p53+/+) cells for 8 days with increasing concentrations of T1-44. D) A colony formation assay in U2OS (p53+/+) cells was performed for 8 days after treatment with increasing concentrations of T1-44 (i); corresponding IC50 plot is presented (ii). E) IC50 plots from MTT assays performed after treating U2OS (p53+/+) cells for 8 days with increasing concentrations of T1-44. F) An immunoblot demonstrating SDMe levels in T47D (p53-/-) cells after 6 days of treatment with increasing concentrations of T1-44 (i) and T1-68 (ii); actin included as a loading control. G) IC50 plots from MTT assays performed after treating T47D (p53-/-) cells for 8 days with increasing concentrations of T1-44 and T1-68.

Fig. S3. Genome-wide effects of PRMT5 inhibition dependent on E2F1. A) Venn diagrams showing the overlap of genes up- or down-regulated over 2-fold change (adjusted P value threshold < 0.01) in each treatment condition with respect to DMSO treated p53-/- HCT116 cells, filtered for genes containing an E2F1 motif in their proximal promoter region (−900 to +100) based on ENCODE. The data were generated from three independent biological samples. (see also Datasets S1-S3). B) Heatmap of differentially expressed E2F1 target genes (adjusted P value ≤ 0.01, 2-fold change) in each treatment condition with respect to DMSO treated p53-/- HCT116 cells. 2LogFoldchange expression values were converted to the Z-score. Increased expression levels are indicated with darker red colouring, whilst decreased expression levels are indicated with darker blue colouring. Ivory colour represents no significant change (see also Datasets S1-S3). C) Significantly over-represented KEGG pathways (i) and GO terms (ii) encompassing cellular components associated with significant differences in expression upon T1-44 treatment (as compared to DMSO control) in p53-/- WT E2F1 HCT116 cells. Heatmaps show PGSEA statistic (Z-score) which characterize how much the mean of the fold-changes for genes in a certain pathway or gene set deviates from the mean observed in all the genes between T1-44 treatment and the control group. Blue indicates gene sets with decreased expression; while red corresponds to those with increased expression. The row labelled ‘GS’ displays the colour range corresponding to the minimum and maximum value of the statistic. D) As above, but KEGG pathways (i) and GO terms (ii) encompassing cellular components are associated with significant differences in expression upon E2F1 Cr knockdown.

Fig. S4. Confirmation that focal adhesion-related genes are E2F1 targets. An E2F1 ChIP was performed in p53+/+ (i) and p53-/- (ii) WT E2F1 and E2F1 Cr HCT116 cell lines treated for 48h with 1 μM T1-44 or DMSO control. Immunoprecipitated chromatin was analyzed using primers spanning the E2F DNA binding site in the CTTN promoter (marked in red) (iii), along with primers surrounding the known E2F motif in the promoter of CDC6. Primers recognising the actin gene were used as a negative control. An immunoblot is included to demonstrate input protein levels for E2F1, p53 and SDMe (iv); actin included as a loading control. n = 3.

Fig. S5. Focal adhesion-related genes are potential E2F1 targets. Presentation of ENCODE ChIP-seq data for CTTN (i), FLII (ii), AIF1L (iii), and ITGA3 (iv) gene promoters performed using the Genome Browser bioinformatics webtool.

Fig. S6. Cortactin regulates motility by the PRMT5-E2F1 axis control. A) Migration assay performed in WT E2F1 p53+/+ HCT116 cells in the presence or absence of CTTN siRNA, after 2 days of treatment with 1 µM T1-44 or DMSO control. The rate of migration was determined by analysing the slope of the line between 20 and 40 hour intervals, and is presented in the bar charts to the right. n = 3. B) An invasion assay was performed under identical conditions as described above. The rate of invasion was determined by analysing the slope of the line between 20 and 40 hour intervals, and is presented in the bar charts to the right. n = 3; C) An adhesion assay was performed under identical conditions as described above. The rate of adhesion was determined by analysing the slope of the line between 0 and 0.5 hour intervals, and is presented in the bar charts to the right. n = 3. D) An immunoblot demonstrating input protein levels for CTTN, p53 and SDMe for the experiments in A, B and C is displayed; actin included as a loading control.

Fig. S7. The PRMT5-E2F1 axis controls cell motility. A) Migration assay performed in p53+/+ WT E2F1 and p53+/+ E2F1 Cr HCT116 cells after 2 days of treatment with 1 µM T1-44 or DMSO control. The rate of migration was determined by analysing the slope of the line between 30 and 40 hour intervals, and is presented in the bar charts to the right. n = 3. B) An invasion assay was performed under conditions identical to those described above. The rate of invasion was determined by analysing the slope of the line between 30 and 40 hour intervals, and is presented in the bar charts to the right. n = 3. C) An adhesion assay was performed under conditions identical to those described above. The rate of adhesion was determined by analysing the slope of the line between 0 and 0.5 hour intervals, and is presented in the bar charts to the right. n = 3. D) An immunoblot demonstrating input protein levels for E2F1, p53 and SDMe for the experiments in A, B, and C is displayed; actin included as a loading control E) Adhesion assay performed in p53+/+ WT E2F1 and p53+/+ E2F1 Cr HCT116 cells in the presence of CTTN siRNA. The rate of adhesion was determined by analysing the slope of the line between 0 and 0.5 hour intervals, and is presented in the bar charts to the right. n = 3. F) Immunoblot displaying input protein levels for CTTN, E2F1 and p53 for the experiment in E is presented; actin included as a loading control.

Table S1. Additional information about use compounds: T1-44 and T1-68.
