# Supplementary material for: PRMT5 promotes cancer cell migration and invasion through the E2F pathway
Source: Cell Death Dis. 2020 Jul 24;11(7):572. doi: 10.1038/s41419-020-02771-9 (PMC7382496; doi:10.1038/s41419-020-02771-9)
Supplement: Supplementary file 9 — Supplementary Table S1 [file 41419_2020_2771_MOESM9_ESM.docx]

Table S1. Additional information about use compounds: T1-44 and T1-68

| Compound name | Structure | Molecular weight |
| --- | --- | --- |
| T1-44 | 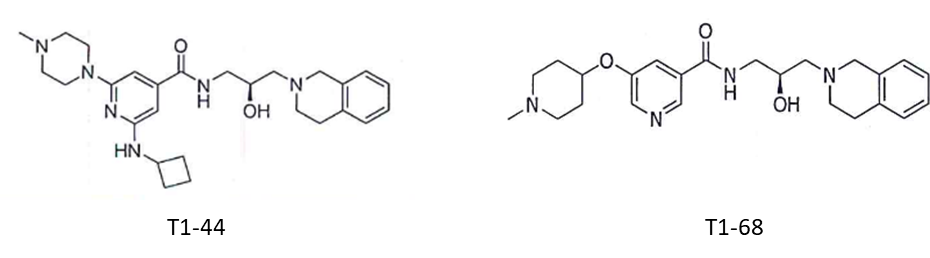 | 478.63 |
| T1-68 | 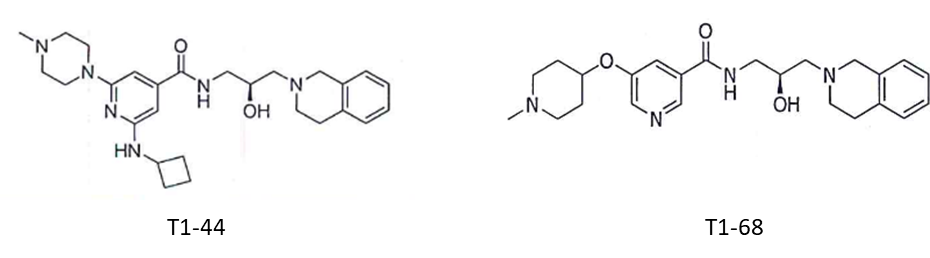 | 424.54 |
